# Pulmonary artery blood flow dynamics in chronic thromboembolic pulmonary hypertension

**DOI:** 10.1038/s41598-023-33727-6

**Published:** 2023-04-20

**Authors:** Hideo Tsubata, Naohiko Nakanishi, Keiichi Itatani, Masao Takigami, Yuki Matsubara, Takeshi Ogo, Tetsuya Fukuda, Hitoshi Matsuda, Satoaki Matoba

**Affiliations:** 1 Department of Cardiovascular Medicine, Graduate School of Medical Science, Kyoto Prefectural University of Medicine, 465 Kajii-cho Kawaramachi-Hirokoji, Kamigyo-ward, Kyoto, 602-8566 Japan; 2 Department of Cardiovascular Surgery, Nagoya City University Graduate School of Medical Sciences, Nagoya, Japan; 3 Department of Cardiovascular Medicine, National Cerebral and Cardiovascular Center, Osaka, Japan; 4 Department of Radiology, National Cerebral and Cardiovascular Center, Osaka, Japan; 5 Department of Vascular Surgery, National Cerebral and Cardiovascular Center, Osaka, Japan

**Keywords:** Vascular diseases, Thromboembolism

## Abstract

Chronic thromboembolic pulmonary hypertension is caused by incomplete resolution and organization of thrombi. Blood flow dynamics are involved in thrombus formation; however, only a few studies have reported on pulmonary artery blood flow dynamics in patients with chronic thromboembolic pulmonary hypertension. Furthermore, the effects of treatment interventions on pulmonary artery blood flow dynamics are not fully understood. The aim of the study was to evaluate pulmonary artery blood flow dynamics in patients with chronic thromboembolic pulmonary hypertension before and after pulmonary endarterectomy and balloon pulmonary angioplasty, using computational fluid dynamics. We analyzed patient-specific pulmonary artery models of 10 patients with chronic thromboembolic pulmonary hypertension and three controls using computational fluid dynamics. In patients with chronic thromboembolic pulmonary hypertension, flow velocity and wall shear stress in the pulmonary arteries were significantly decreased, and the oscillatory shear index and blood stagnation volume were significantly increased than in controls. Pulmonary endarterectomy induced redistribution of pulmonary blood flow and improved blood flow dynamics in the pulmonary artery. Balloon pulmonary angioplasty improved pulmonary blood flow disturbance, decreased blood flow stagnation, and increased wall shear stress, leading to vasodilatation of the distal portion of the pulmonary artery following balloon pulmonary angioplasty treatment.

## Introduction

Chronic thromboembolic pulmonary hypertension (CTEPH) is categorized as group 4 pulmonary hypertension (PH) with poor prognosis^1–3^. The number of patients with CTEPH has been increasing^4^. Although CTEPH is closely related to pulmonary thromboembolism (PE)^5–7^, the etiology and mechanisms of progression are not well understood. It is commonly assumed that CTEPH is caused by incomplete resolution and organization of the thrombus, leading to occlusive pulmonary vascular remodeling^6^. Blood flow stagnation, hypercoagulability, and vascular endothelial dysfunction are all involved in thrombus formation. However, the detailed mechanisms of the development of CTEPH remains unclear.

Recently, simulations using computational fluid dynamics (CFD) have been introduced in clinical medicine, making it possible to visualize blood flow and evaluate detailed blood flow dynamics such as wall shear stress (WSS), which indicates mechanical stress in the vascular endothelium, flow velocity, and energy loss using a patient-specific 3D model^8–10^. In the systemic circulation, it has been reported that low WSS induces endothelial dysfunction, oxidative stress, inflammatory cell adhesion, blood stagnation, and increased lipid uptake, leading to advanced coronary atherosclerosis with plaque rupture^11^. However, little is known about the mechanism by which blood flow dynamics in the pulmonary artery affect disease progression in patients with CTEPH.

Recent improvements in treatment options have led to changes in treatment strategies for CTEPH. Patients with surgically accessible obstructions can undergo pulmonary endarterectomy (PEA), which is a potentially curative treatment that improves functional outcomes and demonstrates a high survival rate^12,13^. Moreover, balloon pulmonary angioplasty (BPA) is also used worldwide^14,15^ because it improves symptoms, exercise tolerance, right heart function, and long-term prognosis^14,16^. Despite improvements in treatment options for the management of CTEPH^17^, the effects of these interventions on pulmonary artery blood flow dynamics, blood stagnation, and pulmonary vascular remodeling are not fully understood.

In this study, we evaluated pulmonary artery blood flow dynamics in patients with CTEPH using patient-specific models. In addition, we explored the effects of PEA and BPA on pulmonary artery blood flow dynamics and pulmonary vascular remodeling.

## Methods

### Study participants

Ten patients with CTEPH and three controls were included in the study. Patients with CTEPH were classified into central and peripheral types according to the surgical classification by the University of California, San Diego^18^. The central thrombus type was classified as Level I and Level II disease, and the peripheral type was classified as level III or IV disease. Five central patients were treated with PEA between November 2016 and June 2018. BPA was performed in five patients with peripheral type CTEPH from December 2015 to August 2018. The control group included healthy individuals who underwent computed tomography (CT) for suspected atrial septal defects, but did not have any abnormalities including atrial septal defects. ECG-gated computed tomography pulmonary angiography (CTPA) with 0.5 mm slice was performed on all patients with CTEPH before treatment. Follow-up CTPA was performed 3–6 months after treatment. This study was approved by the Institutional Review Board of the Kyoto Prefectural University of Medicine (ERB-C-1157). All methods were carried out in accordance with the relevant guidelines and regulations, and with the declaration of Helsinki. The need for informed consent was waived by the research ethics board due to the retrospective nature of the study.

### Geometries and meshes

**Figure 1:**
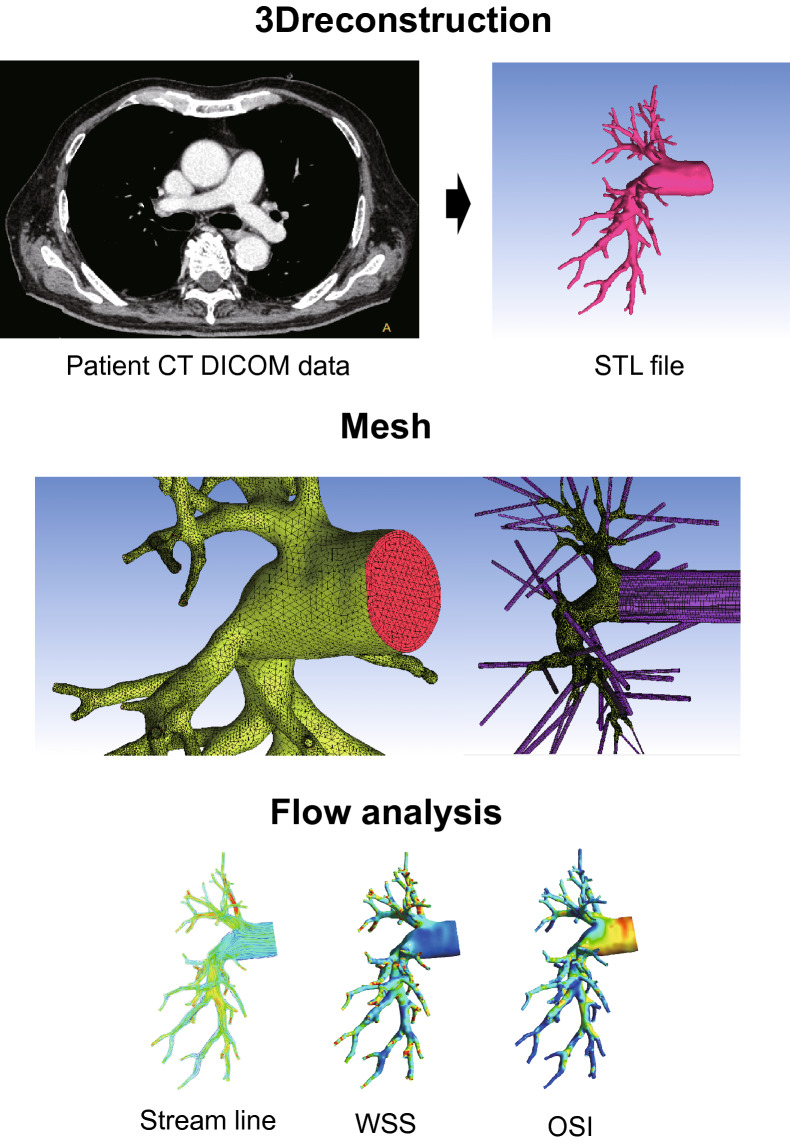
Work process of analysis using computational fluid dynamics. 3D geometries of patient-specific pulmonary arteries in control individuals and patients with CTEPH were generated using computed tomography pulmonary angiography before and after PEA or BPA. After the tetrahedral meshes and boundary-fitted prism layers were created, a CFD simulation was performed to reproduce patient-specific pulmonary artery flow dynamics. CTEPH, chronic thromboembolic pulmonary hypertension; PEA, pulmonary endarterectomy; BPA, balloon pulmonary angioplasty. CT, computed tomography; DICOM, digital imaging and communications in medicine; STL, stereolithography; WSS, wall shear stress; OSI, oscillatory shear index.

The details of the computational analysis methods have been previously described (Fig. 1)^8,19–21^. Data for analyses were acquired using CTPA. The PA geometry is extracted by the semiautomatic segmentation in deep learning trained medical image workstation Ziostation 2 (Ziosoft Inc., Tokyo, Japan) based on threshold method for binarization of CTPA images, and the accuracy was checked with measurement of diameter in several representative points in each case. For computational mesh generation, we used the 3D-Coat software (Pilgway, Kyiv, Ukraine) to make smooth surface, and even afterward check the diameters above to avoid underestimation of vessel volume caused by surface smoothing. The independent observer evaluated the accuracy of the 3D construction. Due to the limitation of CT resolution, it was difficult to construct 3D images of the peripheral area beyond the subsegmental arteries less than 3 mm in diameter. In this study, we assumed that the lumen was completely open, with the wall of the canal through which the contrast agent flowed as the boundary. The constructed geometric sizes were assessed using accurate measurements from the original DICOM files. Computational meshes were created using ANSYS-ICEM CFD 16.2 software (ANSYS, Inc. Japan; Tokyo, Japan). We generated more than 2,000,000 cells with tetrahedral meshes (a minimum of 0.5 mm and a maximum of 5 mm) and three boundary-fitted prism layers^20,22^. Prism layers were created at the boundaries to calculate the WSS near the wall. In patients with CTEPH, peripheral pulmonary artery blood flow stagnates because of an organized thrombus, resulting in insufficient contrast perfusion. Therefore, we limited the vessel sizes to those that could be filled with a mesh. When calculating the hemodynamics of the distal pulmonary artery branches, we limited the analysis to the right pulmonary artery because the analysis on both pulmonary arteries would be a complicated calculation process due to the numerous branches (Supplementary Fig. 1).

### Boundary conditions

To avoid uncertainties from boundary conditions and realize physiological pulsatile flow in the pulmonary arteries, the inlet boundaries of the right main pulmonary artery were extended in the upstream direction to five times their diameters to develop the velocity profiles formed in the boundary layer according to our previously described method^8–10,20^. The inlet boundary conditions in the right main pulmonary artery were set as mass flow boundary conditions with a pulsatile wave. Because it was difficult to accurately determine the blood flow volume through the right pulmonary artery, we measured cardiac output by the thermodilution method using a Swan-Ganz catheter, indicating pulmonary blood flow volume, and set at half of it by assuming that it flowed equally into the left and right pulmonary arteries. In order to simulate the peripheral capacitance, the outlet boundaries for the segmental pulmonary arteries were extruded in downstream direction to 50 times the diameter of each vessel to obtain a stable flow split into branches for sufficient blood pressure recovery at each branch^20^. The outlet boundary conditions were defined as pressure boundary conditions that reflected external forces from outside the analysis area. Pulmonary artery pressure was measured by the Swan-Ganz catheter. One of the main external forces is the reflection wave. The following formula was used to determine the reflection:

1 $$ P_{{{\text{measured}}}} - Z_{0} Q_{{{\text{inlet}}}} $$

where *P*_measured_ is the measured pressure wave and *Q*_inlet_ is the total inlet flow. *Z*_0_ is the characteristic impedance of the pulmonary artery estimated from the pressure and flow wave measurement data. Pulmonary artery walls were assumed to be rigid. To determine the inertial properties of the vessel wall, we set the inertial term with inductance L to maintain the intravessel pressure with the flow change, estimated with the calculated results with the simple linear flow^9,10,21^, and the following term is added to the pressure outlet boundary condition:

2 $$-\mathrm{L}\frac{\mathrm{dQinlet}}{\mathrm{dt}}$$

The assumption of reflection wave outlet boundary conditions including peripheral reflection and vessel inertance for all outlet pulmonary arteries as described previously^23^ with inlet proximal defined flow volume are considered to be feasible to realize the hemodynamics in these PA system.

### Turbulent pulsatile flow simulations

To accurately reproduce patient-specific pulmonary artery flow dynamics, CFD simulation was performed using hemodynamics from right heart catheterization and pulmonary artery geometry from CTPA before and after treatment intervention. The ANSYS-FLUENT 16.2 software (ANSYS, Inc.) was used to solve the Navier–Stokes equation of an incompressible transient Newtonian fluid. The blood density was set to 1060 kg/m^3^ and the blood viscosity was 0.004 kg/m/s. Because the Reynolds number was approximately 4000 in the peak systolic phase, the turbulent flow simulation was applied using the RNG k-ε turbulent model, based on our previous method applied to large vessels^9,10,21,24^. Each time step was set to 10–5 s in the transient flow simulation to sufficiently reduce the Courant number. The convergence criterion was set to 10-5 times the residual for all degrees of the parameters at each time step.

### Wall shear stress, oscillatory shear index, and stagnation volume

Based on the calculated results, the flow velocity, WSS, and oscillatory shear index were evaluated at the peak systolic phase. The WSS is a vector with force and direction toward the vessel wall at a certain point in time. The OSI is an index of the fluctuation of the WSS in one cardiac cycle, and is defined as follows:

3 $$\mathrm{OSI}=\frac{1}{2}\left(1-\frac{\left|{\int }_{0}^{T}\overrightarrow{WSS}dt\right|}{{\int }_{0}^{T}\left|\overrightarrow{WSS}\right|dt}\right)$$

These parameters were averaged from circumferentially at least 10 points on the pulmonary artery walls at the midpoints of each pulmonary artery branch as much as possible to avoid variation. The pulmonary artery is a low flow velocity system; therefore, we set the stagnation volume in the pulmonary artery and defined it as velocity < 0.01 m/s^25^.

### Statistical analysis

Continuous variables were assessed for normality. Normal variables are reported as means ± standard deviations, whereas, non-normal variables are reported as medians (interquartile ranges). We used a non-parametric test (Mann–Whitney U test or Wilcoxon signed-rank test) because of the small sample size. Parameters calculated from CFD were averaged circumferentially from 10 points on the pulmonary artery walls at the midpoints of each pulmonary artery lobar branch as much as possible to avoid variation. Similarly, 32 branches treated with BPA were assessed (averaged circumferentially from five points in each branch) to evaluate the direct effect of BPA on pulmonary blood flow dynamics using a linear mixed effects model. Statistical significance was set at p < 0.05. All statistical analyses were performed using the IBM SPSS Statistics software version 22.

## Results

### Pulmonary artery blood flow dynamics in patients with chronic thromboembolic pulmonary hypertension

We compared the pulmonary artery blood flow dynamics of 10 patients with CTEPH with those of three control individuals, to evaluate the influence of blood flow in CTEPH. Table 1 shows the baseline characteristics of the patients with CTEPH. Hemodynamic analysis demonstrated that the mean pulmonary artery pressure (mPAP) was elevated to 44.5 ± 9.5 mmHg and cardiac index (CI) was decreased to 2.1 ± 0.4 L/min, resulting in increased pulmonary vascular resistance (PVR) as 12.2 ± 4.6 WU.

**Table 1:** Baseline characteristics of the study population.

	All CTEPH (n = 10)	Central (n = 5)	Peripheral (n = 5)	p-value
Age, years	66.8 ± 9.3	65.0 ± 9.3	68.6 ± 8.3	0.39
Female, n (%)	8 (80)	3 (60)	5 (100)	0.11
WHO-FC I/II/III/IV, n	0/1/6/3	0/0/3/2	0/1/3/1	0.51
6MWD, m	401.7 ± 54.9	369.0 ± 53.1	442.5 ± 16.3	0.02
BNP, pg/mL	207.5 (93.2–252.0)	209.0 (157.0–220.0)	206.0 (23.8–252.0)	0.75
Medication, n (%)				
sGC stimulator	1 (10)	1 (20)	0 (0)	0.29
Diuretics	7 (70)	5 (100)	2 (40)	0.03
Hemodynamics				
RAP, mmHg	6.7 ± 5.1	2.4 ± 1.5	11.0 ± 3.0	< 0.01
PAWP, mmHg	9.0 ± 4.5	5.8 ± 2.7	12.2 ± 3.7	0.01
sPAP, mmHg	79.3 ± 18.3	86.2 ± 21.3	72.4 ± 13.2	0.29
dPAP, mmHg	26.5 ± 7.0	26.2 ± 8.5	26.8 ± 6.1	0.67
mPAP, mmHg	44.5 ± 9.5	46.6 ± 12.1	42.4 ± 6.7	0.46
CO, L/min	3.2 ± 0.7	3.0 ± 0.4	3.5 ± 0.8	0.17
CI, L/min/m^2^	2.1 ± 0.4	2.1 ± 0.3	2.2 ± 0.5	0.75
PVR, WU	12.2 ± 4.6	14.3 ± 5.7	10.0 ± 2.0	0.20

Data are presented as n (%), mean ± SD, or median (interquartile range). WHO-FC, World Health Organization functional class; 6MWD, 6-min walk distance; BNP, brain natriuretic peptide; sGC, soluble guanylate cyclase; RAP, right atrial pressure; PAWP, pulmonary artery wedge pressure; sPAP, systolic pulmonary artery pressure; dPAP, diastolic pulmonary artery pressure; mPAP, mean pulmonary artery pressure; CO, cardiac output; CI, cardiac index; PVR, pulmonary vascular resistance.

**Figure 2:**
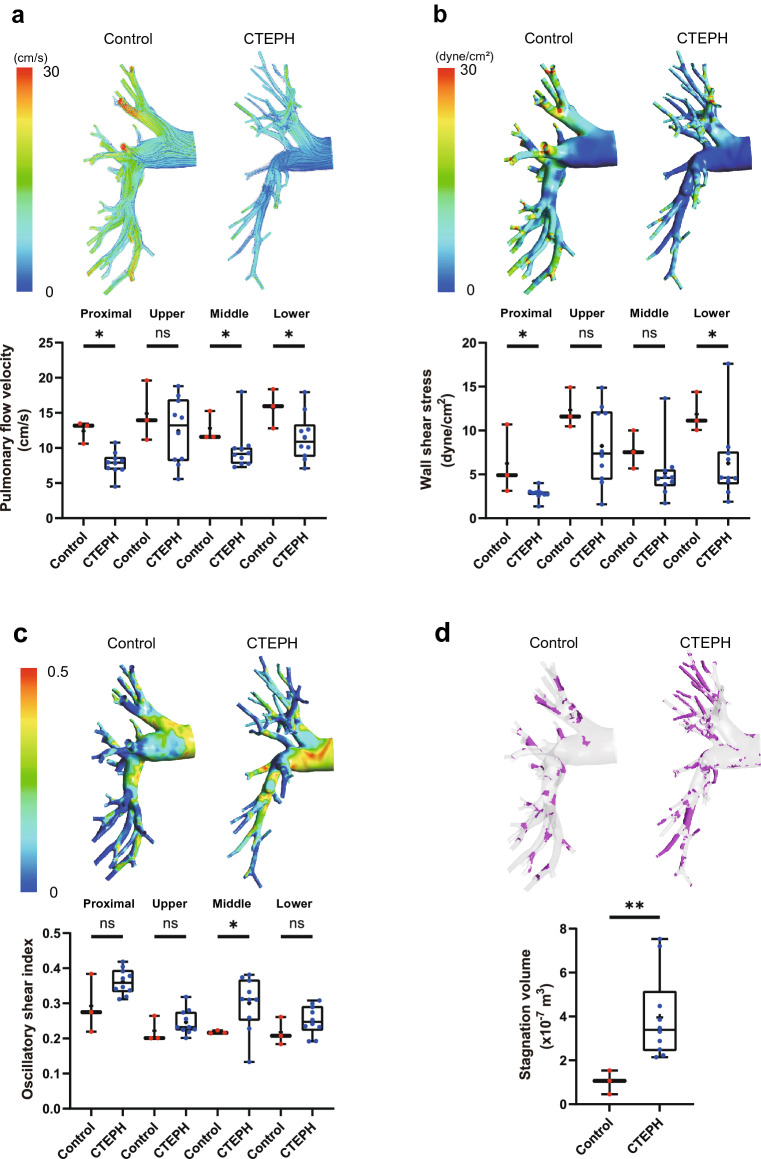
Comparison of pulmonary blood flow dynamics between control individuals and patients with CTEPH. Pulmonary flow velocity (**a**), wall shear stress (**b**), oscillatory shear index (**c**), and blood stagnation volume (**d**) were simulated by CFD in the pulmonary arteries (proximal pulmonary artery, upper lobe branch, middle lower branch, and lower lobe branch of the right pulmonary artery) of control individuals and patients with CTEPH. Stagnation was defined as a flow velocity < 0.01 m/s. Data are presented as the median (interquartile range). Cross indicates mean. The numbers in each box plot indicate the sample size for each group. *p < 0.05 compared to control individuals; ns, not significant; CTEPH, chronic thromboembolic pulmonary hypertension.

Figure 2 and Supplementary Table 1 show the pulmonary artery blood flow dynamics between the control individuals and patients with CTEPH. In the pulmonary artery of patients with CTEPH, flow velocity was significantly lower than that of control individuals (Fig. 2a, Supplementary videos S1 and S2). In addition, the WSS in patients with CTEPH was significantly attenuated compared to that in control individuals (Fig. 2b, Supplementary videos S3 and S4). The oscillatory shear index (OSI) indicates the degree of WSS fluctuation during the cardiac cycle; therefore, the OSI increases in the intima where the turbulence disturbs the WSS direction in a cardiac cycle. The OSI is an important hemodynamic parameter associated with increased radical oxygen species in endothelial cells^26,27^. In the pulmonary artery of patients with CTEPH, the OSI increased compared with that in the control individuals (Fig. 2c). In accordance with these results, there was a greater degree of blood stagnation in the pulmonary artery of patients with CTEPH than in controls (Control, 1.02 ± 0.54 × 10^–7^ m^3^; CTEPH, 3.97 ± 1.94 × 10^–7^ m^3^, p < 0.01) (Fig. 2d, Supplementary videos S5 and S6).

### Comparison of pulmonary artery blood flow dynamics between central and peripheral type chronic thromboembolic pulmonary hypertension

To assess the effect of the thrombus lesion site on pulmonary artery hemodynamics, we compared blood flow dynamics in patients with central and peripheral type CTEPH. The baseline characteristics of the two groups are shown in Table 1. There were no significant differences in mPAP, CI, and PVR between central and peripheral-type CTEPH. Patients with central type CTEPH had lower right atrial pressure and pulmonary artery wedge pressure.

**Figure 3:**
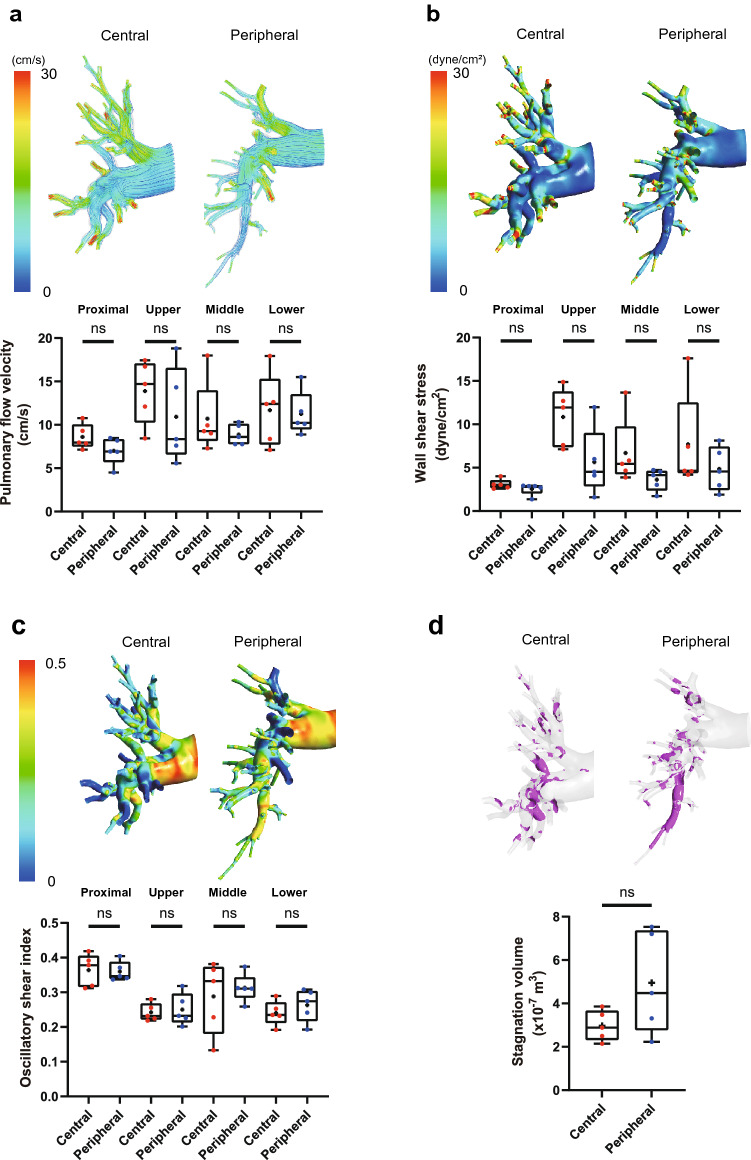
Comparison of pulmonary blood flow dynamics between central and peripheral type CTEPH. Pulmonary flow velocity (**a**), wall shear stress (**b**), oscillatory shear index (**c**), and blood stagnation volume (**d**) were simulated by CFD in the pulmonary arteries of patients with CTEPH in comparison with the central and peripheral types. Stagnation was defined as flow velocity of < 0.01 m/s. Data are presented as median (interquartile range). Cross indicates mean. The numbers in each box plot indicate the sample size for each group. ns, not significant.

When assessing pulmonary artery hemodynamics using CFD, we found that pulmonary flow velocity, WSS, and OSI were comparable between patients with central and peripheral CTEPH (Fig. 3a–c, and Supplementary Table 2). Patients with peripheral type CTEPH tended to have a larger stagnation volume than those with central type CTEPH, but no significant difference was observed (central, 2.97 ± 0.70 × 10^–7^ m^3^; peripheral, 4.95 ± 2.35 × 10^–7^ m^3^; p = 0.22) (Fig. 3d). These results suggest that the thrombus lesion site seems to be independent of the flow velocity, WSS, and OSI in the pulmonary artery of patients with CTEPH.

### Improvement and redistribution of pulmonary blood flow dynamics after pulmonary endarterectomy

**Figure 4:**
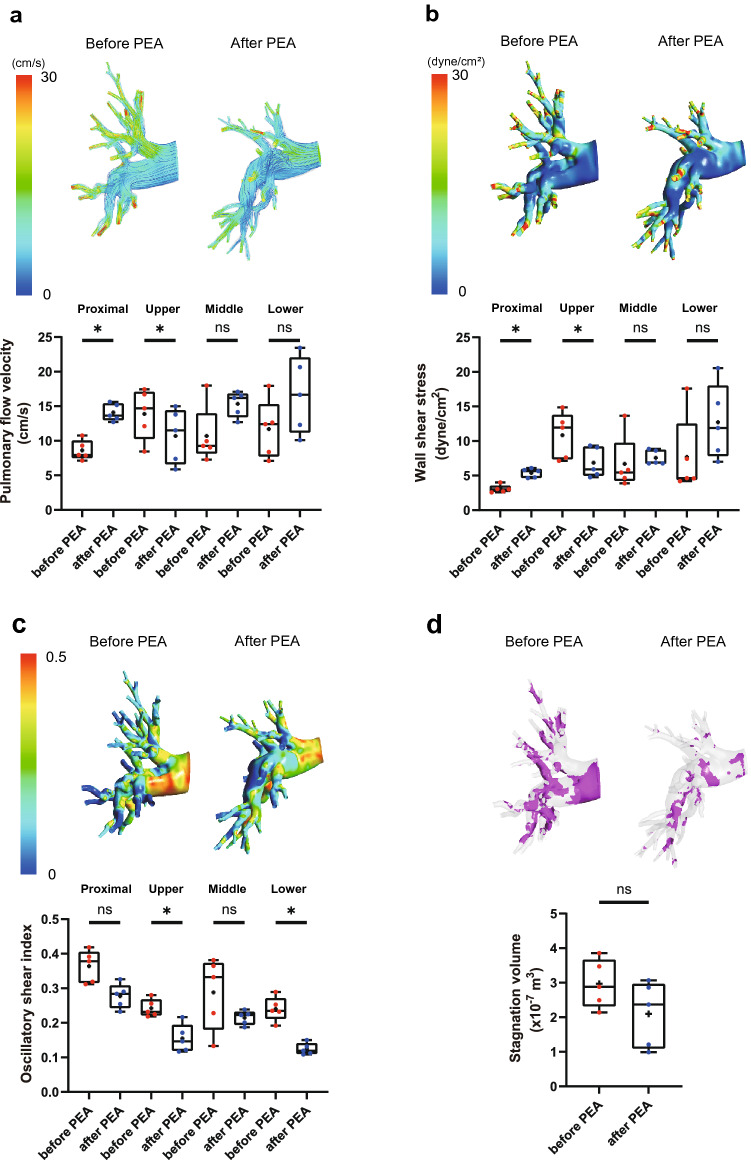
Pulmonary blood flow dynamics before and after PEA. Pulmonary flow velocity (**a**), wall shear stress (**b**), oscillatory shear index (**c**), and blood stagnation volume (**d**) were simulated using CFD in the pulmonary arteries of patients in comparison of before and after PEA. Stagnation was defined as a flow velocity < 0.01 m/s. Data are presented as median (interquartile range). Cross indicates mean. The numbers in each box plot indicate the sample size for each group. *p < 0.05 compared to before PEA; ns, not significant; PEA, pulmonary endarterectomy.

Supplementary Table 3 shows the characteristics and hemodynamics of patients with CTEPH treated with PEA. PEA significantly improved the hemodynamics and symptoms. Flow velocity and WSS were significantly increased in the proximal portion of the pulmonary artery after PEA (Fig. 4a,b, Supplementary Table 4, and Supplementary videos S7, S8, S9, and 10). In contrast, the upper lobe branch showed a significant reduction in flow velocity and WSS. These results suggest that high blood flow volume in the pulmonary artery branches without thrombotic lesions before PEA was redistributed to the branches in which the organized thrombus was resected after PEA, and this relative decrease in pulmonary blood flow volume resulted in the vascular steal phenomenon^28,29^. Moreover, the OSI was significantly decreased in both the upper and lower lobe branches, and tended to be reduced in other regions (Fig. 4c). Although PEA did not reduce the total blood stagnation volume in the pulmonary artery (Before PEA, 2.97 ± 0.70 × 10^–7^ m^3^; after PEA, 2.10 ± 0.95 × 10^–7^ m^3^; p = 0.22) (Fig. 4d, Supplementary videos S11 and S12), the area of blood stagnation in the proximal portion of the pulmonary artery decreased after PEA. Therefore, these results indicate that PEA induces redistribution of pulmonary blood flow and improves blood flow dynamics in the proximal portion of the pulmonary artery.

### Improvement of blood flow dynamics and thrombogenicity of the pulmonary artery after balloon pulmonary angioplasty

**Figure 5:**
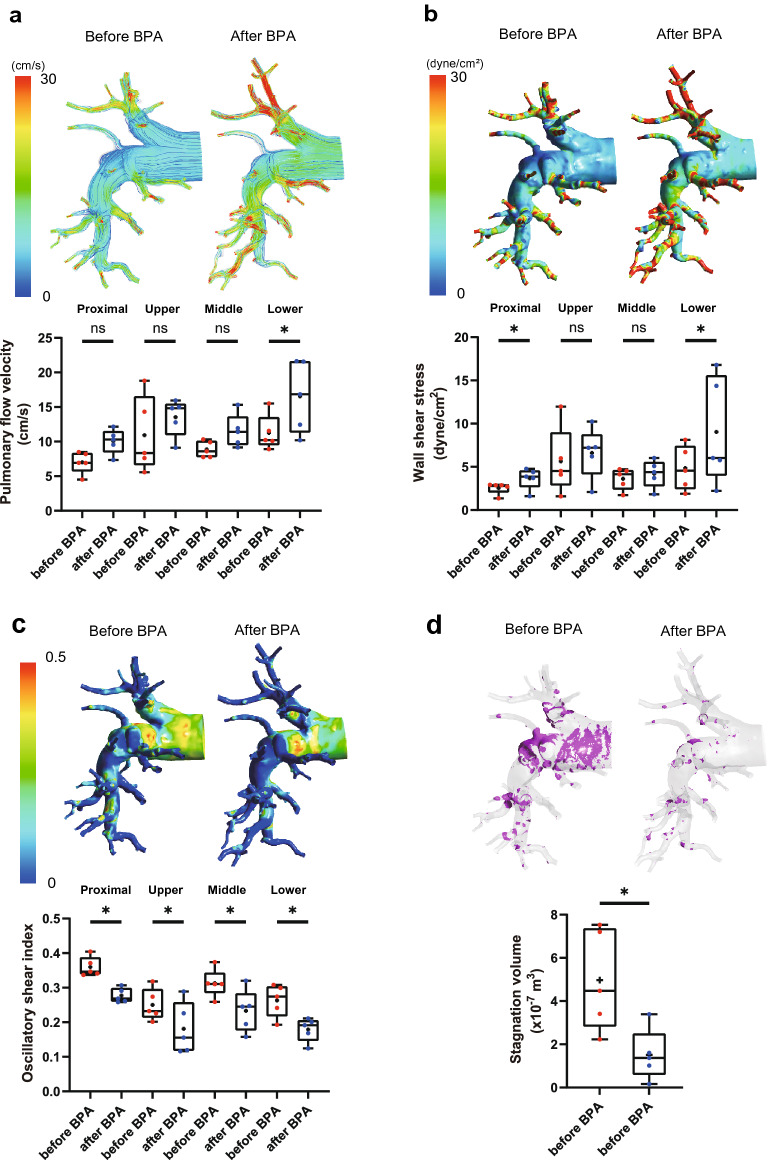
Pulmonary blood flow dynamics before and after BPA. Pulmonary flow velocity (**a**), wall shear stress (**b**), oscillatory shear index (**c**), and blood stagnation volume (**d**) were simulated by CFD in the pulmonary arteries of patients in comparison of before and after BPA. Stagnation was defined as flow velocity of < 0.01 m/s. Data are presented as the median (interquartile range). Cross indicates mean. The numbers in each box plot indicate the sample size for each group. *p < 0.05 compared to before BPA; ns, not significant; BPA, balloon pulmonary angioplasty.

Subsequently, we analyzed pulmonary blood flow dynamics after BPA to evaluate the impact of BPA on pulmonary blood flow and determine the differences between PEA and BPA. Supplementary Table 5 shows the characteristics and hemodynamics of patients with CTEPH treated with BPA. The flow velocity in the lower lobe branches of the pulmonary artery was accelerated, and the WSS in the proximal and lower lobe branches was significantly increased after BPA (Fig. 5a,b, Supplementary Table 6, and Supplementary videos S13, S14, S15, and S16). Although we performed BPA on 8.0 ± 1.1 branches in the right pulmonary artery, improvement in blood flow was remarkable in the lower lobe branch, suggesting the superiority of BPA in the lower lobe branches due to the larger size of the physiological compartment in the vascular bed. Nevertheless, OSI was significantly decreased in all pulmonary arteries after BPA treatment (Fig. 5c). In addition, the blood stagnation volume in the pulmonary artery was significantly decreased after BPA (Before BPA, 4.97 ± 2.33 × 10^–7^ m^3^; after BPA, 1.51 ± 1.19 × 10^–7^ m^3^, p = 0.04) (Fig. 5d, Supplementary videos S17 and S18). These data suggest that BPA not only reduces pulmonary artery pressure, but also improves pulmonary blood flow disturbance, increases WSS, and decreases OSI in the pulmonary artery.

### Local blood flow analysis in the pulmonary artery treated by balloon pulmonary angioplasty

**Figure 6:**
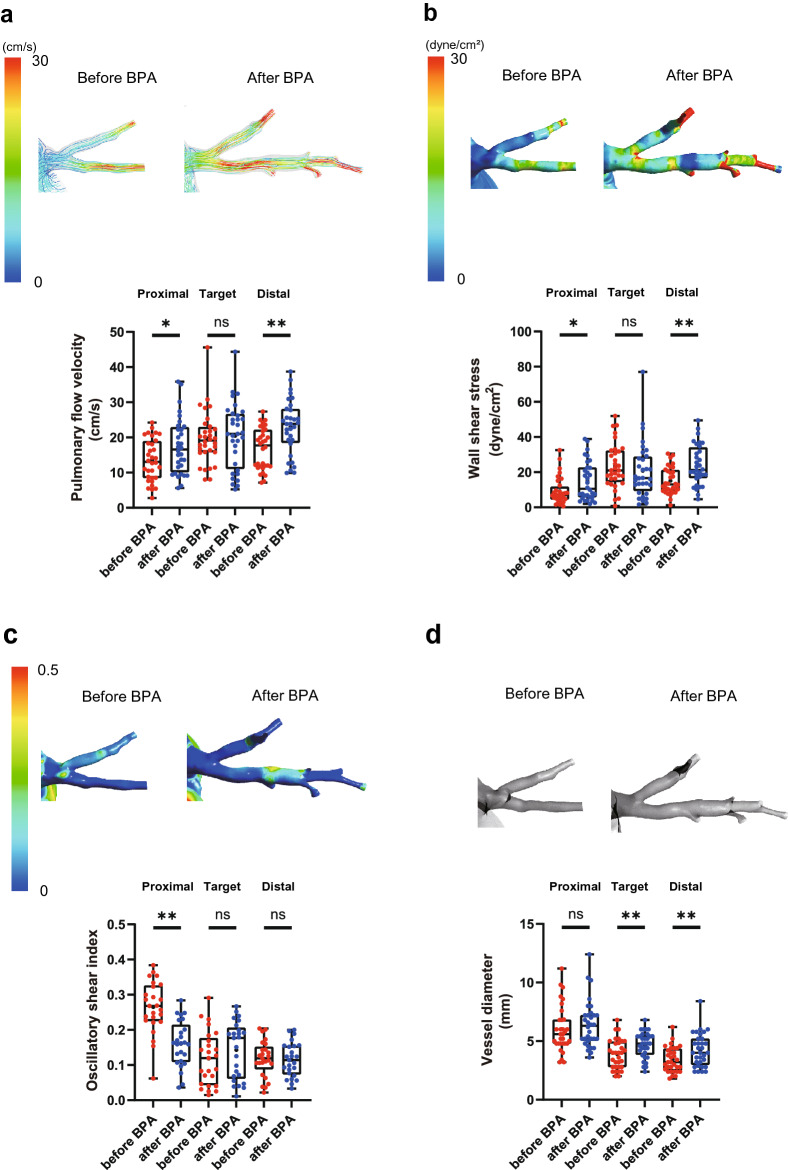
Local pulmonary blood flow dynamics in the pulmonary artery treated by BPA. Pulmonary flow velocity (**a**), wall shear stress (**b**), and oscillatory shear index (**c**) were simulated using CFD in the proximal portion, target lesion, and distal portion of the pulmonary artery branches treated with BPA. (**d**) The vessel diameters of the pulmonary arteries were measured before and after BPA. Data are presented as the median (interquartile range). Cross indicates mean. The numbers in each box plot indicate the sample size for each group. *p < 0.05 compared with before BPA; **p < 0.01 compared with before BPA; ns, not significant; BPA, balloon pulmonary angioplasty.

To evaluate the direct effect of BPA on pulmonary artery hemodynamics, we investigated the local blood flow dynamics in pulmonary artery branches treated with BPA (Fig. 6 and Supplementary Table 7). Flow velocity significantly increased after BPA in the proximal and distal portions of the treated site (Fig. 6a, Supplementary videos S19 and S20). WSS was also significantly increased not only in the proximal portion (Before BPA, 9.2 ± 7.7 dyne/cm^2^; after BPA, 14.9 ± 11.0 dyne/cm^2^; p = 0.01) but also in the distal portion of the treated site after BPA (Before BPA, 15.1 ± 7.6 dyne/cm^2^; after BPA, 24.0 ± 11.4 dyne/cm^2^; p < 0.01) (Fig. 6b, Supplementary videos S21 and S22), suggesting improved blood flow after BPA increased shear stress in the distal pulmonary artery. In addition, the abnormally increased OSI in the proximal portion of the treated site was adequately reduced after BPA treatment because of the improvement in the stagnated blood flow (Fig. 6c). Regarding the morphology of the pulmonary artery, vessel diameter was significantly enlarged after BPA (Fig. 6d). BPA efficiently improved pulmonary artery blood flow dynamics.

## Discussion

Although it is assumed that CTEPH is associated with PE^5–7^, the etiology of CTEPH has not been fully elucidated. A recent registry of PE demonstrated that residual thrombi were detected in 74% of patients after anticoagulation therapy for 1 year by using a refined computed tomography imaging method^30^. In contrast, 0.1–9.1% of patients with symptomatic PE develop CTEPH within 2 years^31,32^, while most patients silently progress to CTEPH without acute events. Although inflammation and infection seem to be involved in the cause of CTEPH^7^, the precise mechanism of incomplete clot resolution or development of organized thrombi is still unknown. In this study, we explored pulmonary blood flow dynamics using patient-specific CFD models in patients with CTEPH and found that in addition to the decreased flow velocity and WSS, OSI and blood stagnation were increased in the pulmonary artery of patients with CTEPH, suspected that these may contribute in part to increased thrombogenicity. Furthermore, both PEA and BPA improved pulmonary blood flow dynamics; however, there were some differences in the improvement effect.

It has been reported that blood velocity and WSS simulated with patient-specific computational models decrease in the pulmonary arteries of patients with pulmonary arterial hypertension^33^. Regarding patients with CTEPH, some reports have shown CFD simulations focused on the treatment effect of PEA or BPA on pulmonary artery flow dynamics^34,35^. However, these are simulations in which thrombotic lesions are artificially created and not patient-specific models that consider patient-specific morphology and hemodynamics. To accurately reproduce the patient's pulmonary artery flow dynamics, we performed CFD simulations using hemodynamics from right heart catheterization and pulmonary artery geometry from CT pulmonary angiography before and after the treatment intervention. Our simulation results provide the patients-specific virtual pulmonary artery blood flow in patients with CTEPH.

Some reports have focused on pulmonary flow dynamics and pulmonary vascular remodeling in patients with CTEPH using magnetic resonance imaging (MRI). These studies showed turbulent flow with reflected waves and decreased blood flow velocity in the main pulmonary artery, which recovered after PEA or BPA^36–38^, consistent with our results. However, these reports only evaluated the main pulmonary artery blood flow and not the peripheral pulmonary artery blood flow due to its lower resolution. In our study, we used CFD models to analyze pulmonary artery blood flow dynamics up to the segmental and subsegmental branches, which are the target regions of BPA. Local blood flow analysis showed that flow velocity and WSS significantly increased after BPA. Improvement in blood flow disturbances and increased shear stress leads to endothelial nitric oxide (NO) synthase expression^39^, NO synthesis^40^, and vascular dilatation. Vascular dilatation decreases pulmonary artery pressure and increases pulmonary blood flow, which induces positive feedback for the recovery of endothelial function. These results were consistent with previous reports that the pulmonary artery treated with BPA had a larger diameter on follow-up angiography^41^.

Although the survival rate of patients with CTEPH has improved in recent years, the treatment effects, focusing on pulmonary blood flow dynamics and pulmonary artery remodeling, have not been fully investigated. This study suggests that abnormal mechanical stress caused by turbulent blood flow around an organized thrombus contributes to CTEPH. Both PEA and BPA improved these extraordinary hemodynamics; however, there were some differences between these treatments. PEA mainly improved the blood flow patterns in the proximal portion and lower lobe branch of the pulmonary artery, as well as decreased blood flow and WSS in the upper lobe branches, indicating redistribution of pulmonary blood flow. BPA also improved both the blood flow patterns in the proximal and lower lobe branches of the pulmonary artery, decreased OSI in the pulmonary artery, and stagnation of the pulmonary artery blood flow. However, BPA did not cause a redistribution of blood flow in the pulmonary artery. BPA increased the WSS in the distal portion of the pulmonary artery and decreased thrombogenicity. Since our study was based on a small number of patients, it is not clear whether these differences in pulmonary hemodynamics are due to differences in treatment or distribution of lesions. In addition, disease severity is also associated with pulmonary artery blood flow dynamics. We need a large number of patients to examine changes in pulmonary blood flow dynamics with different treatment modalities.

**Figure 7:**
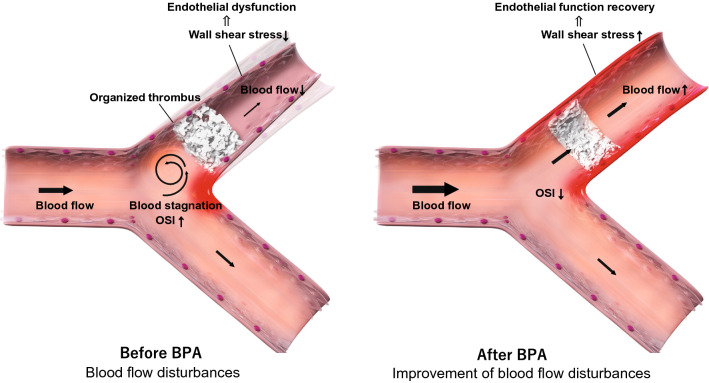
Working hypothesis of pulmonary artery blood flow dynamics before and after BPA.

Our blood flow dynamics analysis showed that turbulence occurred and that blood flow stagnated in the pulmonary artery of patients with CTEPH. Decreased blood flow induces a lower WSS, which may lead to pulmonary artery endothelial cell dysfunction. Increased OSI levels also enhance oxidative stress and chronic inflammation. A combination of these factors can cause thrombogenicity. Treatment with BPA improves WSS in the pulmonary artery, which can lead to an improvement in vascular endothelial cell function and NO production. BPA also reduces OSI and blood stagnation in the pulmonary artery. These reactions lead to the dilation of blood vessels, reduction of blood stagnation (Fig. 7). These findings demonstrate new therapeutic effects of BPA on blood flow dynamics and pulmonary artery reverse remodeling, which have not yet been evaluated.

This study has several limitations. First, this study included only a few patients in two institutes; potential confounders could have influenced the pulmonary blood flow dynamics. Second, this study used simulation data and did not directly measure parameters. In CTEPH, peripheral pulmonary vascular resistance is not considered the same because of the distribution and morphology of thrombotic lesions or microvasculopathy. Thus, our results should be validated using 4D-MRI in the future. Third, this study considered the peripheral reflected waves of the pulmonary arteries and did not consider biological changes in vascular properties due to aging or changes after PEA surgery, which can affect pulmonary artery compliance. Fourth, CFD analysis accuracy is compromised due to incomplete 3D model reconstructions that lacked branches or were terminated prematurely. Our simulations could not provide peripheral artery blood flow since the geometries of pulmonary artery branches are truncated due to CT resolution. These truncated geometries may have affected the calculation results. Fifth, we could not examine the pulmonary artery endothelial cell function and NO production in vivo. Finally, we analyzed the pulmonary blood flow dynamics of patients with CTEPH before and after the intervention. However, these results do not reflect the causes of the disease. Thus, the results of this study suggest that abnormal mechanical stress due to organized thrombi might be involved in the CTEPH, but further investigation is required to elucidate the etiology of CTEPH.

In summary, there is a decrease in blood flow velocity and WSS, and an increase in OSI and blood stagnation in the pulmonary artery of patients with CTEPH. PEA improves blood flow dynamics in the proximal portion of the pulmonary artery and redistributes pulmonary blood flow. BPA improves pulmonary blood flow disturbances and decreases thrombogenicity in the pulmonary artery. Our findings reveal previously undescribed treatment effects of PEA and BPA and provide novel insights into the physiology of CTEPH.

## Supplementary Information

Supplementary Figure 1.

Supplementary Tables.

Supplementary Video S1.

Supplementary Video S2.

Supplementary Video S3.

Supplementary Video S4.

Supplementary Video S5.

Supplementary Video S6.

Supplementary Video S7.

Supplementary Video S8.

Supplementary Video S9.

Supplementary Video S10.

Supplementary Video S11.

Supplementary Video S12.

Supplementary Video S13.

Supplementary Video S14.

Supplementary Video S15.

Supplementary Video S16.

Supplementary Video S17.

Supplementary Video S18.

Supplementary Video S19.

Supplementary Video S20.

Supplementary Video S21.

Supplementary Video S22.

## Data Availability

The datasets used and/or analyzed during the current study are available from the corresponding author upon reasonable request.
