# Supplementary material for: Pulmonary artery blood flow dynamics in chronic thromboembolic pulmonary hypertension
Source: Sci Rep. 2023 Apr 20;13:6490. doi: 10.1038/s41598-023-33727-6 (PMC10119089; doi:10.1038/s41598-023-33727-6)
Supplement: Supplementary file 2 — Supplementary Tables. [file 41598_2023_33727_MOESM2_ESM.docx]

***Supplementary Material***

**Pulmonary artery blood flow dynamics in chronic thromboembolic pulmonary hypertension**

**Hideo Tsubata, MD^1^, Naohiko Nakanishi, MD, PhD^1*^, Keiichi Itatani, MD, PhD^2^, Masao Takigami, MD, PhD^1^, Yuki Matsubara, MD^1^, Takeshi Ogo, MD, PhD^3^, Tetsuya Fukuda, MD, PhD^4^, Hitoshi Matsuda, MD, PhD^5^, Satoaki Matoba, MD, PhD^1^**

*** Correspondence:** Naohiko Nakanishi, MD, PhD: naka-nao@koto.kpu-m.ac.jp

**Supplementary Table 1.** Pulmonary blood flow dynamics in the control group versus CTEPH patients

|  | Control  (n = 3) | CTEPH  (n = 10) | p-value |
| --- | --- | --- | --- |
| Flow velocity (cm/s) |  |  |  |
| Proximal PA | 12.4±1.6 | 7.8±1.7 | 0.01 |
| Upper lobe branch | 14.9±4.3 | 12.4±4.7 | 0.57 |
| Middle lobe branch | 12.8±2.1 | 9.8±3.1 | 0.04 |
| Lower lobe branch | 15.7±2.8 | 11.5±3.3 | 0.04 |
| WSS (dyne/cm^2^) |  |  |  |
| Proximal PA | 6.2±4.0 | 2.8±0.6 | 0.01 |
| Upper lobe branch | 12.3±2.3 | 8.2±4.4 | 0.28 |
| Middle lobe branch | 7.7±2.2 | 5.2±3.2 | 0.07 |
| Lower lobe branch | 11.9±2.3 | 6.3±4.4 | 0.04 |
| OSI |  |  |  |
| Proximal PA | 0.29±0.08 | 0.36±0.04 | 0.21 |
| Upper lobe branch | 0.22±0.04 | 0.25±0.04 | 0.21 |
| Middle lobe branch | 0.22±0.01 | 0.30±0.08 | 0.04 |
| Lower lobe branch | 0.22±0.04 | 0.25±0.04 | 0.28 |
| Stagnation volume (×10^-7^ m^3^) | 1.02±0.54 | 3.97±1.94 | <0.01 |

Data are presented as the mean ± SD. CTEPH, chronic thromboembolic pulmonary hypertension; PA, pulmonary artery; WSS, wall shear stress; OSI, oscillatory shear index.

**Supplementary Table 2.** Pulmonary blood flow dynamics in central versus peripheral type CTEPH

|  | Central  (n = 5) | Peripheral  (n = 5) | p-value |
| --- | --- | --- | --- |
| Flow velocity (cm/s) |  |  |  |
| Proximal PA | 8.6±1.4 | 7.0±1.6 | 0.22 |
| Upper lobe branch | 13.9±3.7 | 10.9±5.5 | 0.31 |
| Middle lobe branch | 10.7±4.2 | 8.9±1.2 | 0.69 |
| Lower lobe branch | 11.7±4.3 | 11.3±2.6 | 1.00 |
| WSS (dyne/cm^2^) |  |  |  |
| Proximal PA | 3.1±0.6 | 2.5±0.7 | 0.31 |
| Upper lobe branch | 10.8±3.4 | 5.6±3.9 | 0.05 |
| Middle lobe branch | 6.7±4.0 | 3.6±1.3 | 0.09 |
| Lower lobe branch | 7.7±5.7 | 4.9±2.6 | 0.42 |
| OSI |  |  |  |
| Proximal PA | 0.36±0.05 | 0.36±0.03 | 1.00 |
| Upper lobe branch | 0.24±0.05 | 0.25±0.05 | 0.84 |
| Middle lobe branch | 0.29±0.11 | 0.31±0.04 | 1.00 |
| Lower lobe branch | 0.24±0.04 | 0.26±0.05 | 0.31 |
| Stagnation volume (×10^-7^ m^3^) | 2.97±0.70 | 4.95±2.35 | 0.22 |

Data are presented as the mean ± SD. CTEPH, chronic thromboembolic pulmonary hypertension; PA, pulmonary artery; WSS, wall shear stress; OSI, oscillatory shear index.

**Supplementary Table 3.** Baseline characteristics of patients with CTEPH before and after PEA

|  | Before PEA  (n = 5) | After PEA  (n = 5) | p-value |
| --- | --- | --- | --- |
| WHO-FC I/II/III/IV, n | 0/0/3/2 | 0/5/0/0 | 0.03 |
| 6MWD, m | 369.0±53.1 | 509.0±36.1 | 0.04 |
| BNP, pg/mL | 209.0  (157.0-220.0) | 26.7  (18.3-60.6) | 0.04 |
| RAP, mmHg | 2.4±1.5 | 2.0±2.0 | 0.68 |
| PAWP, mmHg | 5.8±2.7 | 5.4±2.4 | 0.78 |
| sPAP, mmHg | 86.2±21.3 | 32.0±9.1 | 0.04 |
| dPAP, mmHg | 26.2±8.5 | 11.0±3.2 | 0.04 |
| mPAP, mmHg | 46.6±12.1 | 20.0±7.3 | 0.04 |
| CO, L/min | 3.0±0.4 | 3.7±0.6 | 0.80 |
| CI, L/min/m^2^ | 2.1±0.3 | 2.5±0.4 | 0.13 |
| PVR, WU | 14.3±5.7 | 3.5±1.8 | 0.04 |

Data are presented as numbers, means ± SD, or medians (interquartile ranges). WHO-FC, World Health Organization functional class; 6MWD, 6-minute walk distance; BNP, brain natriuretic peptide; RAP, right atrial pressure; PAWP, pulmonary artery wedge pressure; sPAP, systolic pulmonary artery pressure; diastolic pulmonary artery pressure; mPAP, mean pulmonary artery pressure; CO, cardiac output; CI, cardiac index; PVR, pulmonary vascular resistance.

**Supplementary Table 4.** Pulmonary blood flow dynamics before versus after PEA

|  | Before PEA  (n = 5) | After PEA  (n = 5) | p-value |
| --- | --- | --- | --- |
| Flow velocity (cm/s) |  |  |  |
| Proximal PA | 8.6±1.4 | 14.0±1.3 | 0.04 |
| Upper lobe branch | 13.9±3.7 | 10.7±4.0 | 0.04 |
| Middle lobe branch | 10.7±4.2 | 15.3±1.9 | 0.08 |
| Lower lobe branch | 11.7±4.3 | 16.6±5.6 | 0.08 |
| WSS (dyne/cm^2^) |  |  |  |
| Proximal PA | 3.1±0.6 | 5.4±0.7 | 0.04 |
| Upper lobe branch | 10.8±3.4 | 6.9±2.2 | 0.04 |
| Middle lobe branch | 6.7±4.0 | 7.6±1.1 | 0.50 |
| Lower lobe branch | 7.7±5.7 | 12.7±5.4 | 0.22 |
| OSI |  |  |  |
| Proximal PA | 0.36±0.05 | 0.28±0.04 | 0.08 |
| Upper lobe branch | 0.24±0.03 | 0.15±0.04 | 0.04 |
| Middle lobe branch | 0.29±0.11 | 0.21±0.02 | 0.13 |
| Lower lobe branch | 0.24±0.04 | 0.12±0.02 | 0.04 |
| Stagnation volume (×10^-7^ m^3^) | 2.97±0.70 | 2.10±0.95 | 0.22 |

Data are presented as the mean ± SD. PEA, pulmonary endarterectomy; PA, pulmonary artery; WSS, wall shear stress; OSI, oscillatory shear index.

**Supplementary Table 5.** Baseline characteristics of patients with CTEPH before and after BPA

|  | Before BPA  (n = 5) | After BPA  (n = 5) | p-value |
| --- | --- | --- | --- |
| WHO-FC I/II/III/IV, n | 0/1/3/1 | 1/4/0/0 | 0.07 |
| 6MWD, m | 442.5±16.3 | 490±53.7 | 0.35 |
| BNP, pg/mL | 206.0  (23.8-252.0) | 15.4  (8.5-37.4) | 0.04 |
| RAP, mmHg | 11.0±3.0 | 7.2±1.8 | 0.06 |
| PAWP, mmHg | 12.2±3.7 | 9.0±2.7 | 0.10 |
| sPAP, mmHg | 72.4±13.2 | 41.4±6.6 | 0.04 |
| dPAP, mmHg | 26.8±6.1 | 12.8±3.3 | 0.04 |
| mPAP, mmHg | 42.4±6.7 | 24.6±4.2 | 0.04 |
| CO, L/min | 3.5±0.8 | 4.2±0.9 | 0.04 |
| CI, L/min/m^2^ | 2.2±0.5 | 2.6±0.5 | 0.04 |
| PVR, WU | 10.0±2.0 | 4.0±1.7 | 0.04 |

Data are presented as numbers, means ± SD, or medians (interquartile ranges). WHO-FC, World Health Organization functional class; 6MWD, 6-minute walk distance; BNP, brain natriuretic peptide; RAP, right atrial pressure; PAWP, pulmonary artery wedge pressure; sPAP, systolic pulmonary artery pressure; diastolic pulmonary artery pressure; mPAP, mean pulmonary artery pressure; CO, cardiac output; CI, cardiac index; PVR, pulmonary vascular resistance.

**Supplementary Table 6.** Pulmonary blood flow dynamics before versus after BPA

|  | Before BPA  (n = 5) | After BPA  (n = 5) | p-value |
| --- | --- | --- | --- |
| Flow velocity (cm/s) |  |  |  |
| Right PA | 7.0±1.6 | 10.0±1.8 | 0.08 |
| Upper lobe branch | 10.9±5.5 | 13.5±2.7 | 0.50 |
| Middle lobe branch | 8.9±1.2 | 11.5±2.4 | 0.08 |
| Lower lobe branch | 11.3±2.4 | 16.5±5.2 | 0.04 |
| WSS (dyne/cm^2^) |  |  |  |
| Right PA | 2.5±0.7 | 3.7±1.2 | 0.04 |
| Upper lobe branch | 5.6±3.9 | 6.6±2.9 | 0.22 |
| Middle lobe branch | 3.6±1.3 | 4.2±1.6 | 0.13 |
| Lower lobe branch | 4.9±2.6 | 9.0±6.2 | 0.04 |
| OSI |  |  |  |
| Proximal PA | 0.36±0.03 | 0.28±0.02 | 0.04 |
| Upper lobe branch | 0.25±0.05 | 0.18±0.07 | 0.04 |
| Middle lobe branch | 0.31±0.04 | 0.23±0.06 | 0.04 |
| Lower lobe branch | 0.26±0.05 | 0.18±0.03 | 0.04 |
| Stagnation volume (×10^-7^ m^3^) | 4.97±2.33 | 1.51±1.19 | 0.04 |

Data are presented as the mean ± SD. BPA, balloon pulmonary angioplasty; PA, pulmonary artery; WSS, wall shear stress; OSI, oscillatory shear index.

**Supplementary Table 7.** Local analysis of the pulmonary artery treated by BPA

|  | Before BPA  (n = 32) | After BPA  (n = 32) | p-value |
| --- | --- | --- | --- |
| Flow velocity (cm/s) |  |  |  |
| Proximal portion | 13.5±6.0 | 17.5±8.0 | 0.01 |
| Target lesion | 19.7±7.6 | 20.4±9.3 | 0.69 |
| Distal portion | 16.7±5.9 | 23.0±7.7 | <0.01 |
| WSS (dyne/cm^2^) |  |  |  |
| Proximal portion | 9.2±7.7 | 14.9±11.0 | 0.01 |
| Target lesion | 23.6±12.7 | 20.8±15.8 | 0.38 |
| Distal portion | 15.1±7.6 | 24.0±11.4 | <0.01 |
| OSI |  |  |  |
| Proximal portion | 0.26±0.07 | 0.16±0.06 | <0.01 |
| Target lesion | 0.12±0.08 | 0.14±0.08 | 0.14 |
| Distal portion | 0.12±0.05 | 0.11±0.05 | 0.80 |
| Vessel diameter (mm) |  |  |  |
| Proximal portion | 6.0±2.0 | 6.4±2.0 | 0.40 |
| Target lesion | 3.9±1.3 | 4.7±1.1 | <0.01 |
| Distal portion | 3.4±1.1 | 4.2±1.4 | <0.01 |

Data are presented as the mean ± SD. BPA, balloon pulmonary angioplasty; WSS, wall shear stress; OSI, oscillatory shear index.

**Supplementary video legends**

Video S1: Pulmonary flow velocity in the pulmonary artery of control individuals.

Video S2: Pulmonary flow velocity in the pulmonary artery of patients with CTEPH.

Video S3: Wall shear stress in the pulmonary artery of control individuals.

Video S4: Wall shear stress in the pulmonary artery of patients with CTEPH.

Video S5: Stagnation volume in the pulmonary artery of control individuals.

Video S6: Stagnation volume in the pulmonary artery of patients with CTEPH.

Video S7: Pulmonary flow velocity in the pulmonary arteries before PEA.

Video S8: Pulmonary flow velocity in the pulmonary arteries after PEA.

Video S9: Wall shear stress in the pulmonary arteries before PEA.

Video S10: Wall shear stress in the pulmonary arteries after PEA.

Video S11: Stagnation volume in the pulmonary arteries before PEA.

Video S12: Stagnation volume in the pulmonary arteries after PEA.

Video S13: Pulmonary flow velocity in the pulmonary arteries before BPA.

Video S14: Pulmonary flow velocity in the pulmonary arteries after BPA.

Video S15: Wall shear stress in the pulmonary arteries before BPA.

Video S16: Wall shear stress in the pulmonary arteries after BPA.

Video S17: Stagnation volume in the pulmonary arteries before BPA.

Video S18: Stagnation volume in the pulmonary arteries after BPA.

Video S19: Pulmonary flow velocity in the pulmonary artery branches before BPA.

Video S20: Pulmonary flow velocity in the pulmonary artery branches after BPA.

Video S21: Wall shear stress in the pulmonary artery branches before BPA.

Video S22: Wall shear stress in the pulmonary artery branches after BPA.
